# Exploratory graphical models of functional and structural connectivity patterns for Alzheimer's Disease diagnosis

**DOI:** 10.3389/fncom.2015.00132

**Published:** 2015-11-03

**Authors:** Andrés Ortiz, Jorge Munilla, Ignacio Álvarez-Illán, Juan M. Górriz, Javier Ramírez, Alzheimer's Disease Neuroimaging Initiative

**Affiliations:** ^1^Department Communications Engineering, Universidad de MálagaMálaga, Spain; ^2^Department Signal Theory, Networking and Communications, University of GranadaGranada, Spain

**Keywords:** Gaussian graphical models, sparse inverse covariance, multiple regression, Alzheimer's disease

## Abstract

Alzheimer's Disease (AD) is the most common neurodegenerative disease in elderly people. Its development has been shown to be closely related to changes in the brain connectivity network and in the brain activation patterns along with structural changes caused by the neurodegenerative process. Methods to infer dependence between brain regions are usually derived from the analysis of covariance between activation levels in the different areas. However, these covariance-based methods are not able to estimate conditional independence between variables to factor out the influence of other regions. Conversely, models based on the inverse covariance, or precision matrix, such as Sparse Gaussian Graphical Models allow revealing conditional independence between regions by estimating the covariance between two variables given the rest as constant. This paper uses Sparse Inverse Covariance Estimation (SICE) methods to learn undirected graphs in order to derive functional and structural connectivity patterns from Fludeoxyglucose (18F-FDG) Position Emission Tomography (PET) data and segmented Magnetic Resonance images (MRI), drawn from the ADNI database, for Control, MCI (Mild Cognitive Impairment Subjects), and AD subjects. Sparse computation fits perfectly here as brain regions usually only interact with a few other areas. The models clearly show different metabolic covariation patters between subject groups, revealing the loss of strong connections in AD and MCI subjects when compared to Controls. Similarly, the variance between GM (Gray Matter) densities of different regions reveals different structural covariation patterns between the different groups. Thus, the different connectivity patterns for controls and AD are used in this paper to select regions of interest in PET and GM images with discriminative power for early AD diagnosis. Finally, functional an structural models are combined to leverage the classification accuracy. The results obtained in this work show the usefulness of the Sparse Gaussian Graphical models to reveal functional and structural connectivity patterns. This information provided by the sparse inverse covariance matrices is not only used in an exploratory way but we also propose a method to use it in a discriminative way. Regression coefficients are used to compute reconstruction errors for the different classes that are then introduced in a SVM for classification. Classification experiments performed using 68 Controls, 70 AD, and 111 MCI images and assessed by cross-validation show the effectiveness of the proposed method.

## 1. Introduction

Alzheimer's Disease (AD) is the most common neurodegenerative disease in elderly people, currently affecting more than 40 million people, and its prevalence is expected to be quadrupled by 2050. There is no yet a cure for AD, and medicine has only managed to slow its progress. Early diagnosis becomes, therefore, crucial to treat the disease effectively and may help to develop new drugs. In addition, methods aiming to figure out the neurodegenerative processes involved in the development of AD can provide a better understanding of the disease and the neurophysiological changes produced. Indeed, these constitute an important tool to develop more effective treatments dealing with the early onset and the development of AD. Nevertheless, this still remains a challenge since only mild cognitive symptoms are present in the early stages of the disease and they are similar to those that appear due to the aging process. AD development, however, has been related to structural and functional changes (e.g., cortical thickness and fiber connections) and the identification of both structural and functional network abnormalities can help to understand how functional brain activity deteriorates from anatomical structure and vice-versa.

The improvement of non-invasive imaging systems and their increasing resolution make possible to obtain *in vivo* information about the subjects under study to complement clinical evaluations. Moreover, image analysis along with statistical processing and machine learning techniques allow to exploit disease-related information contained in the images that cannot be manually squeezed. Potentialities of brain imaging for the diagnosis of AD have been explored using functional neuroimaging (Moradi et al., 2015) and structural imaging (Cuingnet et al., 2010; Westman et al., 2011; Chyzhyk et al., 2012; Liu et al., 2012; Termenon and Graña, 2012). The former is aimed at capturing information of biological functions of the brain such as regional cerebral blood flow or glucose metabolism. Radiotracers and tomography imaging techniques such as Single Emission Computerized Tomography (SPECT) or Positron Emission Tomography (PET), are usually employed. Specifically, Fludeoxyglucose Positron Emission Tomography (18F-FDG-PET) has been extensively used for the diagnosis of the AD. The second group focuses on images with structural information such as Magnetic Resonance Images (MRI), which provide anatomical information of brain tissues. In both cases, computer aided diagnosis systems try to identify patterns associated to cerebral neurodegeneration. This way, methods which seek to infer dependence between brain regions are usually based on the analysis of covariance between activation levels in the different areas. This, however, captures pairwise information and may not be able to effectively characterize the interaction of two brain regions working together factoring out the influence of the rest of the regions. Thus, this paper explores the use of partial correlations as mathematical tool applied to the AD study. Partial correlations correspond to the off-diagonal entries of the inverse covariance matrix and thus models based on the inverse covariance, or precision matrix, allow revealing conditional independence between regions by estimating the covariance between two variables given the rest as constant (Pourahmadi, 2013). Additionally, the number of subjects (sample size) is usually not substantially larger than the number of analyzed regions and therefore the traditional maximum likelihood estimation (MLE) method cannot be employed, and sparse computation must be employed instead. Sparse computation fits perfectly in this framework as brain regions usually only interact with a few other areas; i.e., the brain network is naturally sparse (Hilgetag et al., 2002). More specifically, this paper uses Sparse Inverse covariance estimation (SICE), also known as Graphical Models or graphical LASSO (least absolute shrinkage and selection operator), which allows to reach reliable estimation of the inverse covariance even when the sample size is close or even less than the number of brain regions. Additionally, the SICE methods allow to control the number of zero entries in the matrix (i.e., the sparseness of the inverse covariance estimation) by means of a regularization parameter. This regularization parameter can be further seen as a measure for the strength of correlations between two variables. According to the monotone property of the inverse covariance demonstrated in Huang et al. (2010), weak relationships will disappear earlier than stronger relationships when sparseness increases. The key point is then to observe that the sparsity of the precision matrix of a random vector that follows a multivariate normal distribution, relates to the notion of conditional independence of pairs of variables given the rest. Such dependencies can be displayed by a graph (Gaussian Graphical Model) with vertices corresponding to the components and edges between two vertices are present if and only if the components of these vertices are not conditionally independent (i.e., there is no edge if and only if they are conditionally independent Pourahmadi, 2013).

The exploratory use of partial correlations for the AD study was already shown in Huang et al. (2010), where undirected binary graphs were obtained in order to derive functional connectivity patterns from FDG-PET (which we refer simply as PET hereafter). In this paper, we extend this exploratory work and prove that SICE can also be employed in a discriminative way by means of the residuals in the sparse linear regression. More specifically, the main contributions of this work can be summarized as follows:

- Exploratory Gaussian graphical models are completed introducing the strength of the connections between regions computed from both, functional and structural data. In the case of structural data, patterns are derived from the inter-regional covariation of gray matter (GM) volumes in different areas. Functional and structural models clearly show different connectivity patterns between subject groups, revealing changes in the connections in AD and MCI subjects when compared to Controls. This undoubtedly opens a new way for the study of the AD in the future. This paper also provides some statistical features that support the impressions extracted from the visual inspection of the graphs. These features are chosen to take into account the concept of small-worldness (compromise between segregation and integration), which is assumed to characterize brain networks (Supekar et al., 2008; Rubinov and Sporns, 2010).
- Information provided by the SICE models is used in a discriminative way to classify between classes. Reconstruction errors for the different groups are computed and then used as features that allow separating between classes by means of a Support Vector Machine (SVM). Support Vector Classifiers (SVC) have been used in previous works (e.g., Alvarez et al., 2011 or Ortiz et al., 2013) to classify Alzheimer's disease patients, providing good generalization performance while dealing with the *curse of the dimensionality* problem (Raudys and Jain, 1991). Classification experiments performed using 68 Controls, 70 AD, and 111 MCI images and assessed by cross-validation show the effectiveness of the proposed method. This also serves to confirm that the extracted patterns retain the discriminative information. Finally, regions of interest are derived from the models, by means of the SVM weights used for classification, delineating regions that match with regions indicated in the medical literature.

The rest of the paper is organized as follows. Section 2 shows the methods devised in this work to compute covariation patterns in both, functional and structural data. Section 3 presents the results obtained by applying the previously described methods with the ADNI database. Moreover, this section presents the devised cumulative method, the network graphs computed for each group and eventually, the classification results obtained using the discriminative features extracted from the inverse covariance matrices. These results are compared to results obtained using two baseline methods for extracting features, namely Voxels as Features (VAF) and Principal Component Analysis (PCA). Finally, Section 4 discusses the results presented in the previous section highlighting the main findings along with the conclusions of this work.

## 2. Materials and methods

In this section, the database and the method used in this work for the exploratory analysis as well as the devised method to classify new subjects by means of a supervised learning algorithm are described.

### 2.1. Database

Data used in the preparation of this article were obtained from the Alzheimer's Disease Neuroimaging Initiative (ADNI) database (adni.loni.usc.edu). The ADNI was launched in 2003 by the National Institute on Aging (NIA), the National Institute of Biomedical Imaging and Bioengineering (NIBIB), the Food and Drug Administration (FDA), private pharmaceutical companies and non-profit organizations, as a $60 million, 5-year public-private partnership. The primary goal of ADNI has been to test whether serial magnetic resonance imaging (MRI), positron emission tomography (PET), other biological markers, and clinical and neuropsychological assessment can be combined to measure the progression of mild cognitive impairment (MCI) and early Alzheimer's disease (AD). Determination of sensitive and specific markers of very early AD progression is intended to aid researchers and clinicians to develop new treatments and monitor their effectiveness, as well as lessen the time and cost of clinical trials. The Principal Investigator of this initiative is Michael W. Weiner, MD, VA Medical Center and University of California, San Francisco. ADNI is the result of efforts of many co-investigators from a broad range of academic institutions and private corporations, and subjects have been recruited from over 50 sites across the U.S. and Canada. The initial goal of ADNI was to recruit 800 subjects but ADNI has been followed by ADNI-GO and ADNI-2. To date these three protocols have recruited over 1500 adults, ages 55–90, to participate in the research, consisting of cognitively normal older individuals, people with early or late MCI, and people with early AD. The follow up duration of each group is specified in the protocols for ADNI-1, ADNI-2, and ADNI-GO. Subjects originally recruited for ADNI-1 and ADNI-GO had the option to be followed in ADNI-2. For up-to-date information, see www.adni-info.org.

Specifically, experiments conducted in this work use a subset of FDG-PET and T1-weighted MRI images for 249 subjects, consisting of 68 Normal/control, 111 MCI and 70 AD. It is worth noting that these PET and MRI images are taken at the same examination date and, as explained, only those patients for which both MRI and PET data are available have been selected. In those cases in which multiple examinations from the same patient were available, the first one was selected. Demographic data of patients in the database used in this work are summarized in Table 1. On the other hand, stable MCI patients are used in our experiments, which represent 68 out of 111 of the MCI subjects. Thus, from this point on, the notation MCI refers to stable MCI patients.

**Table 1:** **Demographic data of patients in the multimodal PET/MRI database**.

**Diagnosis**	**Number**	**Age**	**Gender (M/F)**	**MMSE**
Normal (Control)	68	75.81±4.93	43/25	29.06±1.08
MCI	111	76.39±6.96	76/35	26.68±2.16
AD	70	75.33±7.17	46/24	22.84±2.91

### 2.2. Image preprocessing

MRI and PET images from the ADNI database have been spatially normalized according to the PET and VBM-T1 templates, respectively, ensuring that each image voxel corresponds to the same anatomical position. After image registration, all the MRI images from ADNI database were resized to 121 × 145 × 121 voxels with voxel-sizes of 1.5 mm (sagittal) × 1.5 mm (coronal) × 1.5 mm (axial), and PET images were resized to 79 × 95 × 68 voxels with voxel-size of 3 mm (sagittal) × 3 mm (coronal) × 3 mm (axial). Subsequently, MRI and PET images are treated differently. MRI images are segmented into White Matter (WM) and Gray Matter (GM) tissues using the VBM toolbox for SPM (Ashburner, 2011; Structural Brain Mapping Group, 2014). This process, which provides information about GM and WM tissue distributions, is guided by means of tissue probability maps of GM, WM, and cerebro-spinal fluid (CSF). A non-linear deformation field is estimated that best overlays the tissue probability maps on the individual sujects' images. The tissue probability maps provided by the International Consortium for Brain Mapping (ICBM) are derived from 452 T1-weighted scans, which were aligned with an atlas space, corrected for scan inhomogeneities, and classified into GM, WM, and CSF. The segmentation process produces values in the range [0, 1] which denotes the membership probability to a specific tissue. However, only GM images are used in this work.

On the other hand, PET images are also normalized in intensity to compute comparable levels among the images. Intensity normalization is performed by means of the mean image, which is used as a normalization template. Specifically, the normalization value applied to each image is calculated as the mean of the 1% of the voxels with a higher activation level in the template (Alvarez et al., 2011; Padilla et al., 2012). This helps to homogenize the activation levels, using the same scale and making them comparable.

### 2.3. Background on SICE and sparse linear regression

Different studies have tried to characterize the interactions between brain regions (Huang et al., 2009, 2010; Chaves et al., 2011, 2012). Correlation analysis captures pairwise information but it does not factor out the contribution to the pairwise correlation due to global or third-party effects. If this is the goal, partial correlation should be adopted instead. Partial correlations are usually estimated via the maximum likelihood estimation (MLE) of the inverse covariance matrix since partial correlations correspond to the off-diagonal entries of the inverse covariance matrix. MLE, however, only provides reliable estimation if the number of patients (sample size) is considerable higher than the number of regions. Otherwise, it requires the use of methods that use a regularization parameter such as SICE, also known as Gaussian graphical model or graphical LASSO (Pourahmadi, 2013).

Let us assume that *n* samples measured at the *p* selected ROIs on the PET and MRI images are given, and that these data can be reasonably assumed to follow a multivariate Gaussian distribution. That is: *x*_1_, *x*_2_, …, *x*_*n*_ ~ N(μ, Σ), where *x*_*i*_, 1 ≤ *i* ≤ *n*, is a *p*-dimensional vector, μ ∈ ℝ^*p*^ is the mean, and Σ ∈ ℝ^*p*×*p*^ is the covariance. Let Θ = Σ^−1^ be the inverse covariance (or precision) matrix. The empirical covariance is denoted as *S*:

(1) $$\begin{array}{l} S=\frac{1}{n}\overset{n}{\underset{i=1}{\sum }}\left(x_{i}-\mu \right){\left(x_{i}-\mu \right)}^{T} \end{array}$$

It can be derived that the maximum log likelihood estimation of Θ under a multivariate Gaussian model can be obtained as follows:

(2) $$\begin{array}{l} \hat{\Theta }=\underset{\Theta ≻0}{\text{argmax}}\left(\log\left(\det\Theta \right)-tr\left(S\Theta \right)\right), \end{array}$$

where *tr*(*SΘ*) is the trace of (*SΘ*). If *S* was not singular, deriving with regards to Θ and setting it to zero, we would get, as expected, that the maximum likelihood estimate of the inverse covariance is $\hat{\Theta }=S^{-1}$. If *p* > *n*, by contrast, the empirical estimate of *S* becomes singular and a regularization must be applied so that a shrunken estimate of Θ can be obtained through a maximization of the penalized log likelihood function. In this paper we apply the SICE method developed by Huang et al. (2009) that applies the *l*_1_-norm regularization. Thus, SICE finds an estimate for the inverse covariance matrix $\hat{\Theta }$ of the brain regions by solving the following optimization:

(3) $$\begin{array}{l} \hat{\Theta }=\underset{\Theta ≻0}{\text{argmax}}\left(\log\left(\det\Theta \right)-tr\left(S\Theta \right)-\lambda ||\Theta |{|}_{1}\right), \end{array}$$

where || · ||_1_ denotes the sum of absolute values of all the entries in a matrix, and λ > 0 is a pre-selected regularization parameter. When λ is small the constraint has little effect and SICE becomes the usual MLE. Conversely, the larger the values of λ, the more sparse are the estimates for Θ provided by SICE. This is an advantage when trying to extract connectivity models since SICE reports directly on conditional independence between two variables (given the other variables in the multivariate Gaussian distribution). The monotone property proved in Huang et al. (2010) also says that if two brain regions are not connected (there is not a path between them) in the connectivity model at a certain λ, they will never become connected as λ goes larger. This monotone property can be used to derive structural and functional connectivity models for different values of sparseness, corresponding to models with different strength of connections, and analyze their differences for the different groups.

Partial correlation computed with SICE can also be used for classification by using the residual in the sparse linear regression. Given the data *x* = {*f*_1_, *f*_2_, …, *f*_*p*_} measured at the *p* selected ROIs, the *i*-th feature can be estimated by the linear regression as follows:

(4) $$\begin{array}{l} f_{i}=\underset{j\neq i}{\sum }\beta_{ij}f_{j}\;+\;\epsilon_{i},\text{for }i=1,\ldots ,p \end{array}$$

where ϵ_*i*_ is uncorrelated with all variables except *f*_*i*_, and β_*ij*_ measures the relationship between the *i*-th feature and the *j*-th feature given all other features. More specifically,

(5) $$\begin{array}{l} \beta_{ij}=-\frac{\Theta_{ij}}{\Theta_{ii}}, \end{array}$$

with var(ϵ_*i*_) = (1/Θ_*ii*_) and cov(ϵ_*i*_, ϵ_*j*_) = Θ_*ij*_∕(Θ_*ii*_Θ_*jj*_).

For the classification of data into two different classes *A* and *B*, averaged coefficients β_*ij*_ for these classes can be estimated independently from the values given by $\hat{\Theta }$. These coefficients define a matrix for each class: β^*A*^ and β^*B*^ ∈ ℝ^*p*×*p*^. Then, the reconstruction errors ϵ ∈ ℝ^*p*^ are computed for every element *x* ∈ ℝ^*p*^ of the dataset; that is, two error vectors, ϵ^*A*^ and ϵ^*B*^, are computed for every *x*. These reconstruction errors, computed by using β^*A*^ and β^*B*^, respectively, may be high since SICE is better at discovering which entries in the inverse covariance matrix are zero than estimating the exact magnitude of the non-zero entries, but the key observation is that they will show values significantly different depending on the class of *x*. These error vectors are then used to classify using a SVM. For the computation of the matrices β, a voxel selection is accomplished in order to select the most discriminative voxels within each region. This is performed by the Student's two sample *t*-Test with pooled variance estimate. The selected *p*-value is quite low, *p* < 0.01, so that only those voxels whose mean differences show clearly that they do not have statistical relevance are disregarded.

### 2.4. Functional and structural connectivity models, small worldness

A goal of this paper is the characterization of networks composed of brain regions connected by anatomical or functional associations and the establishment of relationships between networks across groups with the aim to reveal presumed connectivity abnormalities. Anatomical connections typically correspond to WM tracts between pairs of brain regions. Although the presence of anatomical connections suggests the potential for functional connections, such connections, may occur between pairs of anatomically unconnected regions. Structural networks are extracted from MRI data, while functional networks are extracted from PET data. Note also that although for clarity we will keep, inherited from Huang et al. (2009), the term “functional” to refer to brain connectivity networks extracted from PET data, these do not correspond to correlation in activity but they, strictly speaking, measure covariation in glucose uptake between different regions, which can be further related to metabolic covariations. Similarly, we use a measure of GM density as structural information, which aims to capture GM inter-regional covariance. This is motivated by the fact that inter-regional GM density covariation is related to the presence of a fiber tract between regions (Segall et al., 2012).

Anatomical and functional connections, to be meaningful, must be defined on the same map of brain regions. We use here the 116-regions Automated Anatomical Labeling Atlas (AAL) to extract the features. Nevertheless, only 42 regions out of 116, distributed in the frontal, parietal, occipital, and temporal lobes, have been selected here for brain connectivity modeling, as they are considered to be potentially related to AD (Huang et al., 2009). Table 2 lists the names of the used regions and includes a number that will be used to index the node in the connectivity models. These regions will be the nodes of our brain networks while links or arcs will be used to denote the presence or absence the connections between nodes. As explained above, they can be seen as an interpretation of the sparse inverse covariance. An arc between two regions represents a non-zero partial correlation and reflects that these two regions are directly connected. Furthermore, if two brain regions are not connected by an arc, but there is a path between them, they can be considered as connected indirectly. For the sake of simplicity, we will adopt a matrix representation for these graphs, with 42 rows and columns corresponding to the regions of interest and a black cell (binarized) indicating the presence of an arc between the corresponding region of interests of that row and column. Since the connectivity graphs are undirected, the matrix is symmetric and the total number of black cells is equal to twice the total number of arcs. The degree of an individual node is equal to the number of links connected to that node, and therefore it reflects the importance of that node. Hub nodes are defined as those with the highest degrees; i.e., with the highest number of edges. A cluster is a group of nodes interconnected among them but isolated from the rest.

**Table 2:** **Names and the corresponding indexes of the regions for connectivity modeling Huang et al. (2010)**.

	**Frontal lobe**		**Parietal lobe**		**Occipital lobe**		**Temporal lobe**
1	Frontal_Sup_L	13	Parietal_Sup_L	21	Occipital_Sup_L	27	Temporal_Sup_L
2	Frontal_Sup_R	14	Parietal_Sup_R	22	Occipital_Sup_R	28	Temporal_Sup_R
3	Frontal_Mid_L	15	Parietal_Inf_L	23	Occipital_Mid_L	29	Temporal_Pole_Sup_L
4	Frontal_Mid_R	16	Parietal_Inf_R	24	Occipital_Mid_R	30	Temporal_Pole_Sup_R
5	Frontal_Sup_Medial_L	17	Precuneus_L	25	Occipital_Inf_L	31	Temporal_Mid_L
6	Frontal_Sup_Medial_R	18	Precuneus_R	26	Occipital_Inf_R	32	Temporal_Mid_R
7	Frontal_Mid_Orb_L	19	Cingulum_Post_L			33	Temporal_Pole_Mid_L
8	Frontal_Mid_Orb_R	20	Cingulum_Post_R			34	Temporal_Pole_Mid_R
9	Rectus_L					35	Temporal_Inf_L 8301
10	Rectus_R					36	Temporal_Inf_R 8302
11	Cingulum_Ant_L					37	Fusiform_L
12	Cingulum_Ant_R					38	Fusiform_R
						39	Hippocampus_L
						40	Hippocampus_R
						41	ParaHippocampal_L
						42	ParaHippocampal_R

*“Functional segregation is the ability for specialized processing to occur within densely interconnected groups of brain regions”* (Rubinov and Sporns, 2010). Hence, the presence of clusters in anatomical networks suggests the potential for functional segregation. On the contrary, functional integration is the ability to combine specialized information from distributed brain regions. Shorter paths imply stronger potential for integration. The term small-world is thought to simultaneously reconcile the opposing demands of functional integration and segregation. A well-designed network should combine an optimal balance of functional integration and segregation, that is, the presence of segregated modules connected (integrated) through links. In SICE analysis, values of the regularization parameter that maximize the number of clusters also likely maximize the small-worldness.

### 2.5. Support vector machines (SVM)

Support Vector Machines (SVM) are a set of supervised learning methods widely used for classification and regression (Vapnik, 1998; Sammut and Webb, 2010), designed to separate a set of binary-labeled data by means of a hyperplane. Specifically, a smart optimization method is used to compute the maximal margin hyperplane so that the maximum separation between classes is achieved. They use a decision function in the form *g*:ℝ^*p*^ → {±1}, corresponding to *p*-dimensional training vectors *x*_*i*_ and class labels *y*_*i*_ with 1 ≤ *i* ≤ *n*:

(6) $$\begin{array}{l} \left(x_{1},y_{1}\right),\left(x_{2},y_{2}\right),\ldots ,\left(x_{n},y_{n}\right)\in {\mathbb{R}}^{p}\times \left\{\pm 1\right\} \end{array}$$

in such a way that *g* is able to correctly classify new samples (*x, y*). The parameter *p* is the dimensionality of the feature vectors.

Linear discriminative functions define decision hyperplanes in a multidimensional feature space:

(7) $$\begin{array}{l} g\left(x\right)=\omega^{T}x+\upsilon_{0} \end{array}$$

where ω is the weight vector and υ_0_ is a bias (threshold). This way, $\omega^{T}x+\upsilon_{0}\geq 1$ if class *y* = +1 and $\omega^{T}x+\upsilon_{0}\leq 1$ if class *y* = −1, being the weight vector ω orthogonal to the decision hyperplane. Finding the optimal separating hyperplane is accomplished by the optimization task of finding the unknown parameters ω and υ_0_ which define the decision hyperplane that separates the two classes optimally. Alternatively, weights assigned to each feature, assigned by the SVM during the optimization process, can be used to rank the features, as explained later on.

### 2.6. Exploratory and classification methods

**Figure 1:**
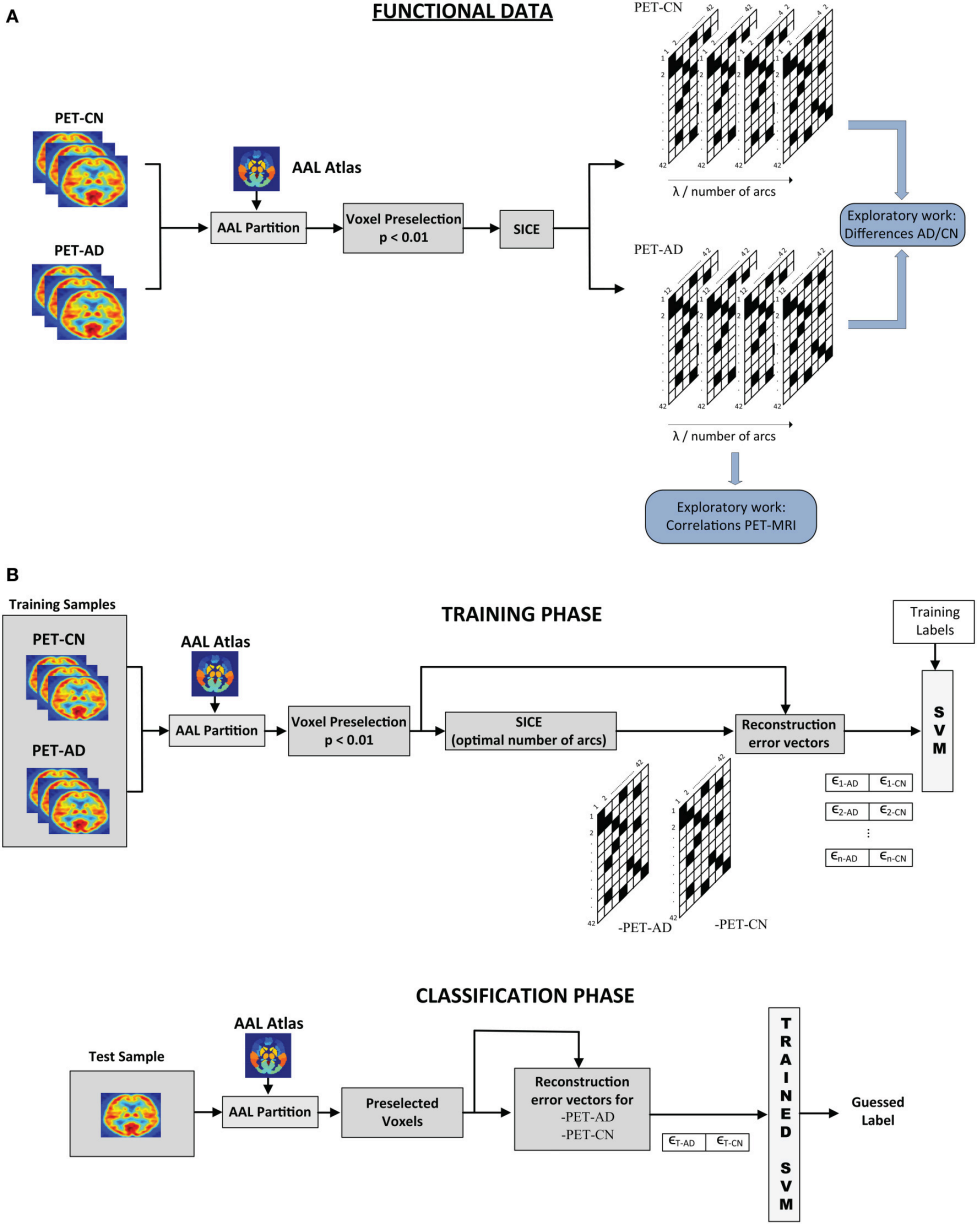
**Scheme of the overall SICE-based process (Functional Data). (A)** Exploratory and **(B)** Classification processes.

The SICE-based analysis presented in this paper comprises an exploratory part, where structural and functional connectivity models are inferred, and a discriminative part, where the connectivity models estimated by SICE enable us to differentiate between CN, MCI, and AD. Figure 1 shows the overall scheme for this analysis: exploratory part (above) and classification process (below). Note that although the procedure is illustrated for functional data of CN and AD, it can be easily adapted for structural data and other group combinations.

For the exploratory work, images are partitioned according to the AAL atlas and the voxel preselection (*p* < 0.01) is performed. SICE is then used to extract functional connectivity models for AD and CN for different values of the regularization parameter. A common strategy (Supekar et al., 2008; Huang et al., 2010) to achieve that the comparison between the different subject groups makes sense is to control the total number of arcs. A sweep of λ is performed until a specific number of arcs is reached. Connectivity models are then computed for a range of total number of arcs.

For classification, once the inverse covariance matrices are estimated for each group, we compute the corresponding regression coefficient. Using these values, the reconstruction error vectors for each image *i* are computed: ϵ_*i*−*CN*_, ϵ_*i*−*AD*_. A support vector machine is then trained using these errors as features. For the test image, the process is similar. Voxels preselected during the training stage are used and the reconstruction vector errors for both CN and AD are computed using the inverse covariance matrix estimated for each group. Such error vectors are then introduced in the trained SVM for classification. When both MRI and PET data are available for classification, error vectors for structural and functional data can be combined in the same SVM. In order to prove that the system is not over-fit and thus, has a good generalization performance, feature selection, and classification processes have been assessed by k-fold cross-validation (*k* = 10). Cross-validation method consists in splitting the sample set into *k* subsets. Then, *k* − 1 subsets are used for training while the classification process is carried out using the excluded subset. These subsets are different and do not share any samples. This process was repeated for the *k*-folds, ensuring test data is never used in the feature selection or the classifier training. This is repeated *k* times, for each fold being used as test sample, and the final result is obtained by averaging. The main purpose of cross-validation is to estimate the generalization error, ensuring that similar results will be obtained on new data (that is, low generalization error). This method estimates the prediction error and avoids double-dipping, providing a lower generalization error variance estimate (Hastie et al., 2003).

## 3. Results

In this section, results obtained from the exploratory analysis using functional (FDG-PET) and structural (MRI) data are shown. These results are presented in two ways. First, by using binarized inverse covariance matrices for different number of arcs that reveal the connectivity between regions for CN, MCI, and AD. And then, through a cluster analysis. This analysis takes into account the strength of the connections and provides an easier graphical way to represent the brain connectivity.

Finally, we demonstrate the capabilities of the partial correlations obtained from SICE for image classification, by computing reconstruction errors using multiple regression analysis.

### 3.1. Functional connectivity analysis from binarized SICE

**Figure 2:**
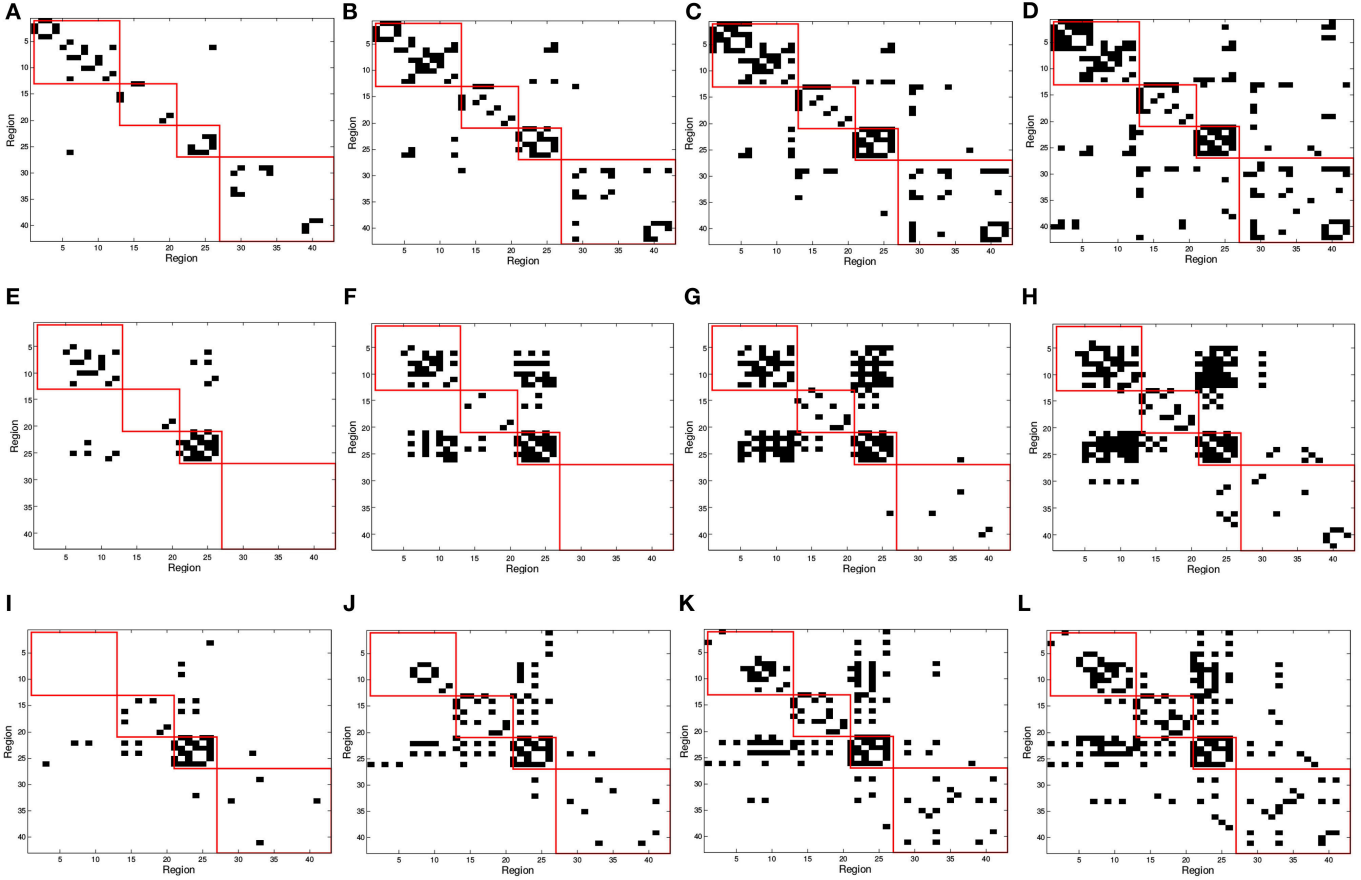
**SICE matrix computed for (A,E,I) 25, (B,F,J) 50, (C,G,K) 75, and (D,H,L) 100 arcs using functional PET data**. First row correspond to CN, second row corresponds to MCI, and third row to AD subjects. Red squares in $\hat{\Theta }$ (from top to bottom, left to right) indicate regions contained in frontal, parietal, occipital, and temporal lobes.

In Figure 2, red squares split the binarized $\hat{\Theta }$ in different brain areas, each including regions from the frontal, parietal, occipital, and temporal lobes. Different connectivity patterns are obtained for the same number of arcs in CN, MCI, and AD patients and these also change differently when varying the number of arcs; i.e., when varying the sparsity parameter λ. The first regards the fact that $\hat{\Theta }$ effectively captures connectivity differences between the group. The latter refers to the strength of the connections; as the number of arcs diminishes (higher λ-values), only the strongest connections remain.

In order to validate statistically the differences between the patterns for the different groups, a distance measure between the corresponding connectivity matrices has been computed. This measure only accounts for those entries which are different (in absolute value) between two matrices with a significance level of 5%. This has been worked out by applying two-sample *t*-test hypothesis testing on the matrix entries computed during k-fold cross-validation (*k* = 10). It is worth noting that only the lower triangular parts of the connectivity matrices (after discarding diagonal elements) have been used to account for the number of different entries as connectivity matrices are symmetric. Table 3 shows the percentage of different entries with *p* < 0.05 in the functional connectivity matrices taken by pairs for CN/AD, MCI/AD, and CN/MCI cases.

**Table 3:** **Number of different entries in the functional connectivity matrices with ***p*** < 0.05**.

**Connectivity matrices**	**Percentage of different entries for**			
	**25 arcs**	**50 arcs**	**75 arcs**	**150 arcs**
CN/AD	70	68	97	71
MCI/AD	62	73	92	68
CN/MCI	56	61	86	61

On the other hand, an analysis of the inter-lobe and intra-lobe connections also allows to draw some conclusions. In general, when the number of arcs increases, and weaker connections are included, the percentages of inter-lobe and intra-lobe connections between the different lobes tend asymptotically to the uniform distribution. For example, occipital lobe represents the 2% of the elements of the matrix. For 10 arcs, inter-lobe connections in the occipital lobe are 20, 60, and 70% for CN, AD, and MCI, respectively. For 225 arcs, these values are 5, 4.9, and 4.4%, approaching to the 2% value. Something similar happens if inter-lobe and intra-lobe connections are analyzed as a whole. Inter-lobe connections are 71% of the elements of the matrix. Nevertheless, for 10 arcs, inter-lobe connections only represent 0, 30, and 0% for CN, AD, and MCI, respectively. For 225 arcs, by contrast, these percentages increase to 53, 57, and 58%. Two findings can be drawn from these results. First, the differences in the number of inter-lobe and intra-lobe connections of the different connectivity networks become more relevant when the number of arcs decreases and only the strongest connections are represented. And second, intra-lobe connections are much stronger than inter-lobe connections. The latter can also serve to determinate differences between the different subject groups. It is particularly interesting to identify differences involving MCI, since literature exists in studying the brain connectivity differences between AD and CN but studies on MCI are limited. For example, intra-lobe connections of the temporal lobe remain almost constant in percentage for CN (about 20%), while they increases from 0% (10 arcs) to about 20% (225 arcs) for AD and MCI. In other cases MCI networks present behaviors similar to CN. For example, although it is a bit higher for CN, the percentage of intra-lobe connections of the frontal lobes shows similar evolution for MCI and CN (decreasing from high values), while it is different for AD (increasing from low values). Something similar occurs with the inter-lobe connections between the parietal and the occipital lobes; they increase for MCI and CN and decrease for AD. These results lead to think that intra-lobe connections of the temporal lobe could be a good indicator of the neurodegenerative process in MCI patients, while others, such as intra-lobe connections in the frontal lobe, do not seem so affected yet at the MCI state.

**Figure 3:**
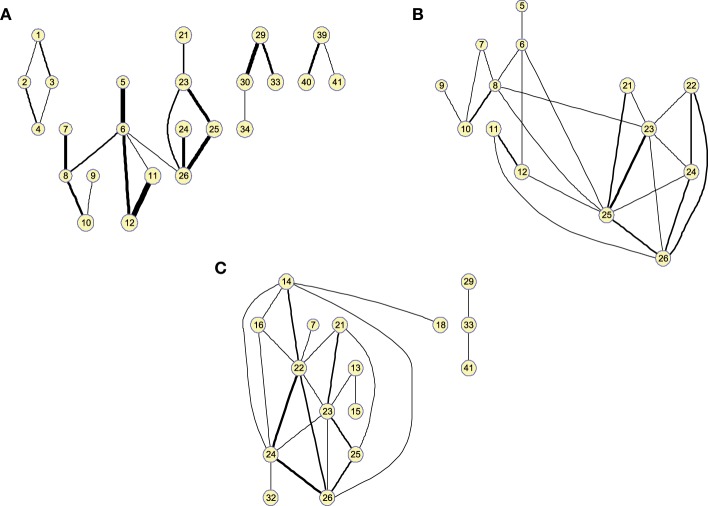
**Clusters found on the brain network computed by functional 25-arc $\hat{\Theta }$ for (A) Controls, (B) MCI, and (C) AD**. Note that isolated nodes are not shown in the graph. Edge thickness represents the normalized strength of the connections.

Matrices $\hat{\Theta }$ can also be represented as graphs for a specific number of arcs. This allows to visually extract information about functional segregation (number of clusters) and integration (hubs). That is, information about the *small worldness* (Supekar et al., 2008; Rubinov and Sporns, 2010) of the brain connectivity network for the different groups. Figure 3 shows the clusters found in the 25-arc $\hat{\Theta }$ computed for CN (a), MCI (b), and AD (c). Different connectivity graphs are clearly identified for each group. While the CN graph seems to exhibit a good balance between integration and segregation, MCI and AD are less segregated with weaker connections (some metrics to support this are given later). The use of 25 arcs has been considered as an optimal trade-off between accuracy and clarity. Thickness of the arc represents the strength of the connections through the normalized absolute values of $\hat{\Theta }$, so that thicker lines correspond to stronger connections. For this specific case, the strength values in CN, MCI, and AD have been uniformly quantized in the range [1, 10] for an easier comparison.

### 3.2. Cumulative SICE for the analysis of the connectivity strength

Matrices $\hat{\Theta }$ are usually binarized. Thus, the strength of the connections is neglected which could cause the loss of valuable information. The monotone property demonstrated in Huang et al. (2010) allows to relate the strength of a connection in a binarized matrix with its resistance to disappear when the sparseness increases; i.e., when λ increases or equivalently the number of arcs decreases. Although strictly speaking this property applies to connections between nodes and not to arcs, both concepts are obviously related. Thus, we devise a method that using as input the “traditional” binarized matrices estimates values for the strength of the connections; i.e., an approximation to the partial correlation coefficients. The obtained values provide consistent results to these obtained directly through the SICE, which allows to gain confidence with the use of these absolute values as measures of the connectivity strength.

The estimated matrix, that we will call cumulative inverse covariance, ${\hat{\Theta }}_{c}$, is computed by the weighted sum of the binarized $\hat{\Theta }$ obtained for different number of arcs. That is:

(8) $$\begin{array}{l} {\hat{\Theta }}_{c}=\underset{n\in narcs}{\sum }\frac{10}{n}bin\left[{\hat{\Theta }}_{n}\right]\;\;\;\;\;narcs=\left\{10,25,50,75,100\right\} \end{array}$$

where $bin\left[{\hat{\Theta }}_{n}\right]$ represents the binarized $\hat{\Theta }$ obtained for *n* arcs. The threshold value used to binarize the matrices $\hat{\Theta }$ has been 0 (i.e., all non-zero entries are considered as 1).

**Figure 4:**
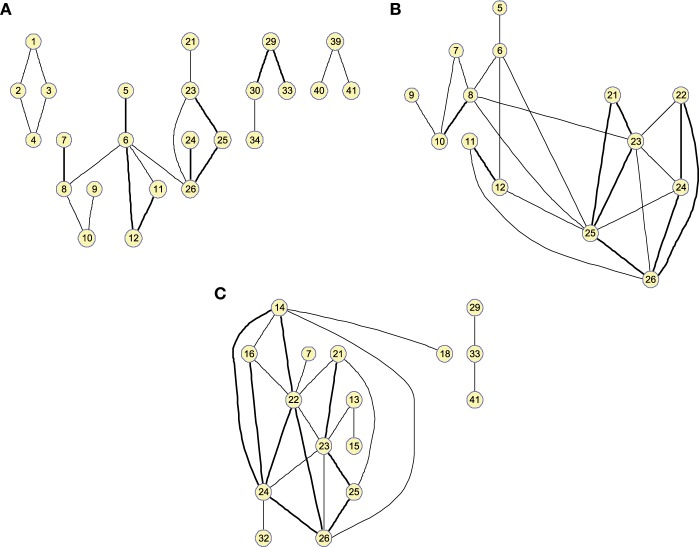
**Twenty-five-arc clusters found on functional ${\hat{\Theta }}_{c}$ computed up to 100 arcs for (A) Controls, (B) MCI, and (C) AD**. Note that isolated nodes are not shown in the graph. Edge thickness represents the normalized strength of the connections.

Figure 4 shows the clusters found on the ${\hat{\Theta }}_{c}$ when the 25 strongest arcs are included. The strength is again indicated by the thickness of the edges connecting each pair of regions. The resulting graphs are very similar to those shown in the previous section, proving that both methods represent effective ways to obtain information about the relative strength of the edges. With ${\hat{\Theta }}_{c}$, however, the connections have a lower resolution and it must also be noted that maximum values for the graphs of the different groups of subjects will have similar values as they are now related to the number of occurrences.

### 3.3. Structural connectivity

In this section, the analysis of the structural network is performed. Information obtained from structural and functional networks is different as they are derived from different sources. In this work, a structural parameter has been derived in order to obtain statistical information for each brain region based on GM density measurements:

(9) $$\begin{array}{l} \rho_{i}=\frac{\#V_{i}^{GM}}{\#V_{i}} \end{array}$$

where $\#V_{i}^{GM}$ is the number of GM voxels in region *i*, and *#V*_*i*_ is the total number of voxels in region *i*.

**Figure 5:**
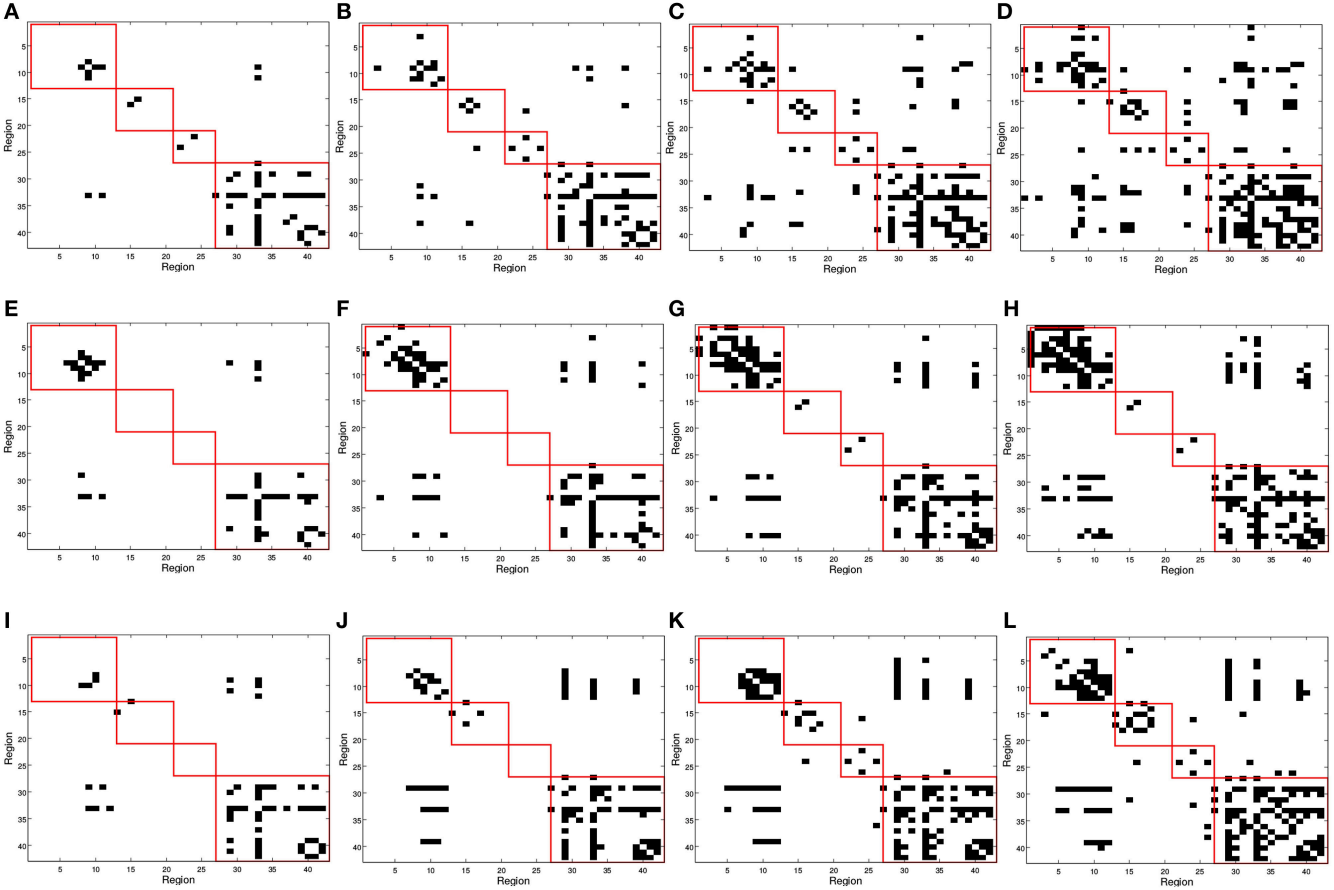
**$\hat{\Theta }$ matrix computed for (A,E,I) 25, (B,F,J) 50, (C,G,K) 75, and (D,H,L) 100 arcs using structural MRI GM information**. First row correspond to Controls, second row corresponds to MCI and third row to AD subjects. Red squares in $\hat{\Theta }$ (from top to bottom, left to right) indicates regions contained in frontal, parietal, occipital, and temporal lobes.

Figure 5 shows the binarized $\hat{\Theta }$ computed using the GM density values ρ_*i*_. In this case, $\hat{\Theta }$ represents inter-regional covariation of GM density, or regions whose GM density level is conditionally independent. This aims to discover structural alterations that could reflect differences in the functionality. The general analysis of inter-lobe and intra-lobes connections shows a similar behavior to that in the functional case. Intra-lobe connections are 71% of the elements of the matrix, but when the number of arcs is reduced they only represent a much smaller percentage; in particular, for 25 arcs, inter-lobe connections represent 7, 16, and 20% for CN, MCI, and AD, respectively. Again, this can be interpreted as a higher strength for the intra-lobe connections than for the inter-lobe connections. Differences can also be observed when comparing different groups; for example, the number of intra-lobe connections between parietal and temporal lobes seems to be higher for CN (0, 2, 4, 8% for 25, 50, 75, and 100 arcs) than for MCI (0, 0, 0, 0%) and AD (0, 0, 0, 1%).

**Figure 6:**
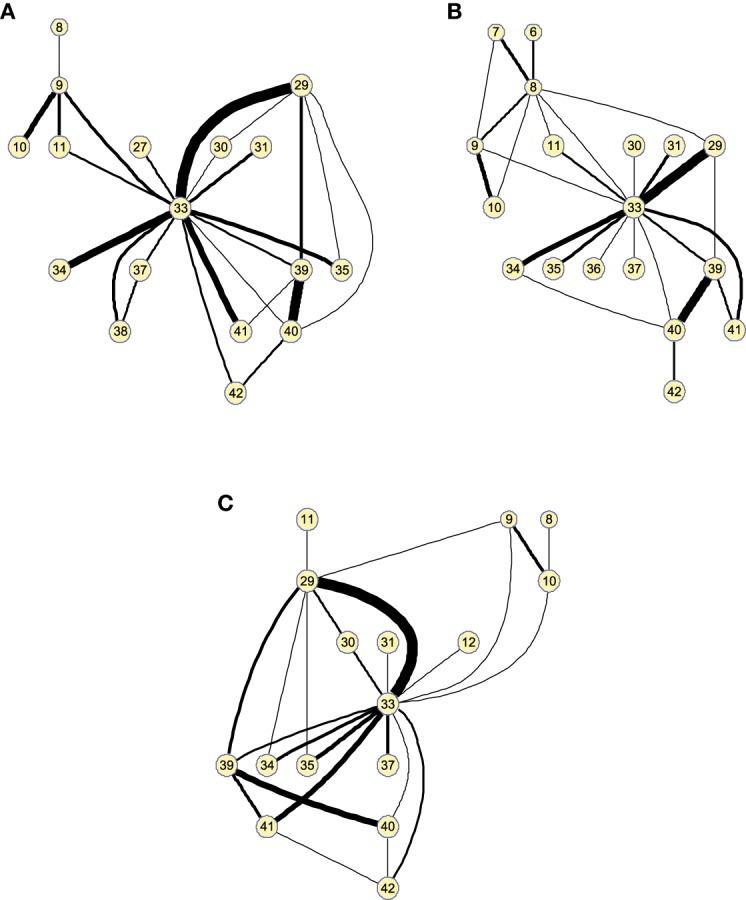
**Small worldness analysis**. Clusters found on the structural brain network computed by 25-arc $\hat{\Theta }$ for **(A)** Controls, **(B)** MCI, and **(C)** AD subjects. Note that isolated nodes are not shown in the graph. Edge thickness represent the normalized strength of the connections (namely covariation).

As before, these results can also be represented as graphs to extract information in a visual fashion. Figure 6 depicts the graphs for 25 arcs; arcs link co-varying regions, while edge widths represent the relative covariance values. Region 33 (Left middle temporal pole) is a hub for the three groups, indicating that its GM density covariation is strongly related to the density covariation of other regions. For 25 arcs, the three graphs, for the three groups, are quite similar and the differences between the graphs lie essentially in the strength of the connections. As we will show in classification, these structural differences between CN and MCI allow a good classification between these groups.

### 3.4. Systematic network analysis

To finish with the exploratory analysis of the networks, a more systematic analysis is carried out by using some of the metrics of graph theory. In particular, the average clustering coefficient and the average number of connections per hub have been chosen for their relationship with the concept of small-worldness, which is supposed to characterize brain networks (Supekar et al., 2008; Rubinov and Sporns, 2010).

1. Average clustering coefficient. This measures the fraction of the node's neighbors that are also neighbors of each other (Watts and Strogatz, 1998). It provides an estimation of network segregation which is related to the number of clusters present in the network and the number of edges interconnecting the nodes in each cluster of the functional network (Rubinov and Sporns, 2010). For functional data, it provides a measure of the network organization that figures out the interconnection of functionally specialized clusters. When computed on the structural $\hat{\Theta }$, it estimates the distribution of regions whose GM density variations are conditionally dependent. The definition of this measure for binary and undirected networks is as follows:
  (10) $$\begin{array}{l} C=\frac{1}{n}\underset{i\in N}{\sum }C_{i}=\frac{1}{n}\underset{i\in N}{\sum }\frac{2t_{i}}{k_{i}\left(k_{i}-1\right)}, \end{array}$$
  where *n* is the number of nodes, *N* is the set of all nodes in the network, and *k*_*i*_, *C*_*i*_, and *t*_*i*_ are the degree, the clustering coefficient (*C*_*i*_ = 0 for *k*_*i*_ < 2) and the number of triangles around the node *i*, respectively. *t*_*i*_ is further computed as:
  (11) $$\begin{array}{l} t_{i}=\frac{1}{2}\underset{j,h\in N}{\sum }a_{ij}a_{ih}a_{jh}, \end{array}$$
  where *a*_*ij*_ is the connection status between *i* and *j*: *a*_*ij*_ = 1 when there exists a link between these nodes; *a*_*ij*_ = 0 otherwise (*a*_*ii*_ = 0 for all *i*).
2. Average number of connections per hub. The number of hub nodes is a measure of functionality distribution. In structural networks, it is a measure of the number of regions whose GM density variation is related to others. The definition of this measure for binary and undirected networks is as follows:
  (12) $$\begin{array}{l} E=\frac{1}{n}\underset{i\in N}{\sum }E_{i}=\frac{1}{n}\underset{i\in N}{\sum }\left(\sum_{j\in N}a_{ij}k_{j}\right) \end{array}$$

**Figure 7:**
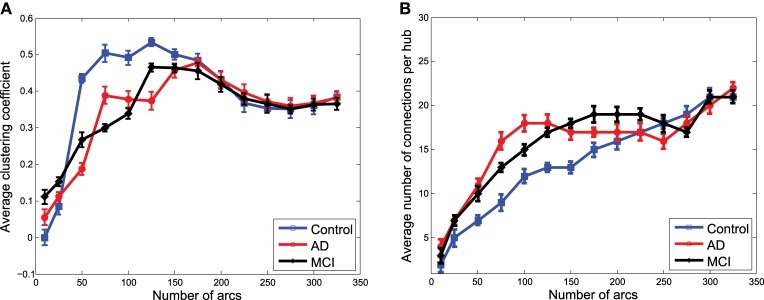
**Average clustering coefficient (A) and average number of connections per hub (B) computed from the clusters in $\hat{\Theta }$ obtained from functional data**.

Figures 7A,B show the average clustering coefficient and the average number of connections per hub, respectively, computed for the functional graphs (see Figure 3). These figures provide a clearer view of the variation profile of network performances when the $\hat{\Theta }$ is computed for a different number of arcs (i.e., different λ-values). The lower clustering coefficient found in CN indicates the trend of CN functional networks to remain more segregated. This is also reflected in the smaller number of connections per hub shown in Figure 7B.

**Figure 8:**
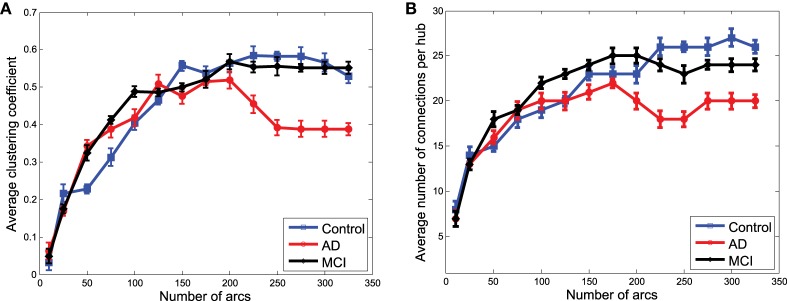
**Average clustering coefficient (A) and average number of connections per hub (B) computed from the clusters in $\hat{\Theta }$ obtained from structural data**.

Network performances (Figure 8) obtained from the clusters for GM structural data also reveal differences between the groups. However, these differences are no longer as relevant as they were for functional data. However, as we will see for classification, when the strength of the connections is taken into account, structural data provide an important discriminative power for the CN/MCI case.

### 3.5. Classification

Different classification experiments have been considered. Firstly, classification tests using only PET data or only GM data are carried out. This allows to compare the discriminative capability of the relationships revealed by the SICE in functional and structural network, respectively. Then, functional and structural features are used jointly by concatenating both error vectors to be used as input to the SVM. As explained in Section 2.6, the results provided here represent the average of the values obtained during the cross-validation process. These results show that, even though partial correlations estimated by SICE may not be very accurate, they can be effectively used for classification by computing linear regression coefficients.

**Figure 9:**
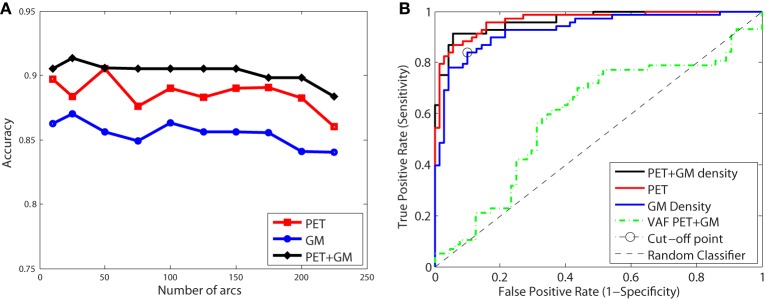
**Accuracy (A) and ROC curve (B) for CN/AD classification considering a different number of arcs in the SICE**.

Figure 9A shows the classification accuracy obtained for CN/AD, resulting in a maximal accuracy of 0.92. Figure 9B depicts the ROC curve which shows an AUC of 0.96. Functional and structural features are not redundant but complementary, as the inclusion of structural features outperforms the results obtained using only functional data. These results also improve the Voxels as Features (VAF) (Stoeckel and Fung, 2005) method used as baseline, which consist in using raw VAF for classification.

**Figure 10:**
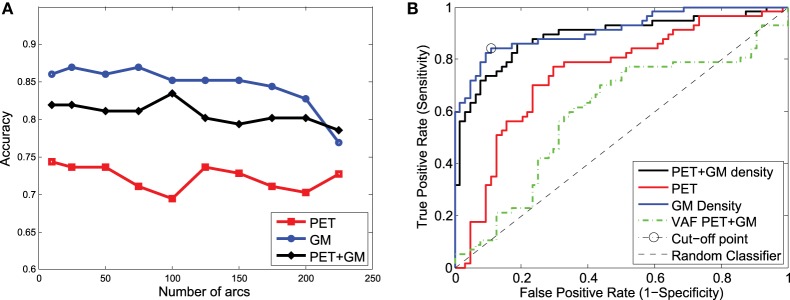
**Accuracy (A) and ROC curve (B) for CN/MCI classification considering a different number of arcs in the SICE**.

These classification experiments have been repeated with MCI/AD and CN/MCI patients. The latter is the most difficult case and the most relevant for early AD diagnosis. For MCI/AD classification, the combination of functional and structural data offers again the best results but, in this case, structural data slightly outperform functional data. For CN/MCI, structural data offer the best results (Figure 10); i.e., they are better than when functional and structural data are used together. This is also shown in the ROC curve (Figure 10B).

Table 4 collects all the classification results. To determine the best performing method the accuracy, sensitivity, specificity, and the Area Under ROC curve (AUC) are provided, along with their standard deviations. AUC takes into account sensitivity and specificity, and it is considered a good metric for classification performance. Additionally, the results have been also assessed by means of ANOVA analysis (Navidi, 2010) using the accuracy values. This analysis, whose results are shown in Table 5, allows to state (in terms of accuracy) whether the methods are providing different accuracy values or not. In this analysis, the null hypothesis (*H*_0_) argues for equal means at 0.05% of significance level. When significant differences were found in the classification accuracy provided by the different methods, a multiple comparison test is performed to reveal the best method. Specifically, the *p*-values obtained for MCI/AD and CN/MCI classification are below 0.05, and therefore, the null hypothesis is rejected at 5% of significance level in both cases. Thus, the average accuracy value provided by our proposal is higher than the ones provided by the VAF or PCA alternatives. For the CN/AD classification, conversely, the *p*-value is above 0.05, and therefore we cannot reject the null hypothesis at five percent level, and thus, the average accuracy values provided by the different methods compared in Table 4 must be considered equal. It is likely, however, according to the AUC values provided by ROC analysis, that SICE methods using PET or PET+GM data perform better than other methods.

**Table 4:** **Classification results**.

**Method**	**Accuracy**	**Sensitivity**	**Specificity**	**AUC**
**CN/AD CLASSIFICATION**				
VAF PET+GM	0.86 ± 0.11	0.85 ± 0.13	0.87 ± 0.16	0.88
PCA PET+GM	0.87 ± 0.10	0.85 ± 0.15	0.90 ± 0.10	0.79
PET SICE regression + SVM	0.90 ± 0.10	0.88 ± 0.14	0.88 ± 0.12	0.96
MRI-GM SICE regression + SVM	0.87 ± 0.09	0.90 ± 0.15	0.84 ± 0.18	0.92
**SICE regression + SVM (PET+GM)**	**0.92 ± 0.05**	**0.96 ± 0.09**	**0.86 ± 0.13**	**0.96**
**MCI/AD CLASSIFICATION**				
VAF PET+GM	0.66 ± 0.11	0.64 ± 0.19	0.69 ± 0.13	0.66
PCA PET+GM	0.70 ± 0.09	0.72 ± 0.11	0.69 ± 0.15	0.77
PET SICE regression + SVM	0.74 ± 0.09	0.72 ± 0.14	0.77 ± 0.17	0.81
MRI-GM SICE regression + SVM	0.80 ± 0.10	0.82 ± 0.13	0.78 ± 0.12	0.86
**SICE regression + SVM (PET+GM)**	**0.84 ± 0.09**	**0.87 ± 0.10**	**0.81 ± 0.12**	**0.88**
**CN/MCI CLASSIFICATION**				
VAF PET+GM	0.62 ± 0.17	0.60 ± 0.27	0.63 ± 0.22	0.59
PCA PET+GM	0.67 ± 0.10	0.66 ± 0.16	0.69 ± 0.11	0.74
PET SICE regression + SVM	0.73 ± 0.15	0.70 ± 0.25	0.77 ± 0.14	0.74
**MRI-GM SICE regression** + **SVM**	**0.86 ± 0.10**	**0.90 ± 0.10**	**0.82 ± 0.18**	**0.91**
SICE regression + SVM (PET+GM)	0.81 ± 0.12	0.83 ± 0.15	0.80 ± 0.18	0.88

*Mean values across the cross-validation iterations are shown along with the standard deviations. Positive class used to compute the statistics is indicated in boldface*.

**Table 5:** **ANOVA analysis results with 5% of significance level**.

**Classification**	***p*-value**	***F*-statistic**	***H*_0_ (Equal means)**
CN/AD	> 0.05	0.56	Not rejected
MCI/AD (SICE-SVM, GM+PET)	0.0016	5.2	Rejected
CN/MCI (SICE-SVM, GM)	0.0013	5.3	Rejected

**Figure 11:**
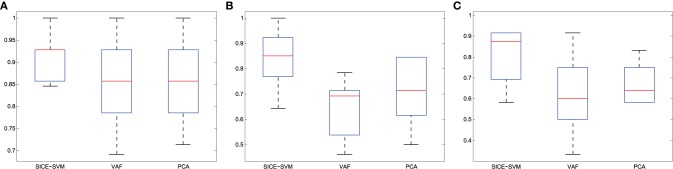
**Accuracy (A) CN/AD, (B) MCI/AD, (C) CN/MCI classification considering the method indicated in bold in Table 4**.

Finally, Figure 11 presents the classification results collected in Table 4 using boxplot graphs. This aims to display differences between groups without making any assumptions about the underlying statistical distribution.

#### 3.5.1. Ranking regions of interest (ROI)

As explained in Section 2.6, a SVM is used to classify the subjects based on the residuals obtained for each region by regressing them using both CN or AD models (or MCI in the cases of CN/MCI and MCI/AD classification). Thus, the most discriminative regions can be found according to their reconstruction errors. The core idea is to select those regions whose reconstruction error is different when reconstructed with Θ^*CN*^ or Θ^*AD*^ (or Θ^*MCI*^). In other words, regions not affected by AD should present similar reconstruction errors when regressed with Θ^*CN*^ or Θ^*AD*^. Thus, detecting the most discriminative ROIs can be carried out by ranking the residuals using the weights that the SVM assigns to each feature. In fact, let *Ns* be the number of *support vectors* within the margin chosen during the training phase, the following vector can be computed:

(13) $$\begin{array}{l} \text{W}=\overset{Ns}{\underset{j=1}{\sum }}y_{j}\lambda_{j}x_{j} \end{array}$$

where *y*_*j*_ are the labels, λ_*j*_ are the corresponding Lagrangian parameters, which are also optimized during the training phase, and *x*_*j*_ are the training samples. The coordinate *i* of the vector *W*, *W*_*i*_ with 1 ≤ *i* ≤ *p*, informs us about the relevance of the *i*-th dimension of the feature vectors (Hidalgo-Muñoz et al., 2014). More precisely, the higher the |*W*_*i*_|, the more the relevance of the *i*-th dimension in the feature vectors. By contrast, |*W*_*i*_| = 0 indicates that the *i*-th feature does not have any influence in the classification process.

**Figure 12:**
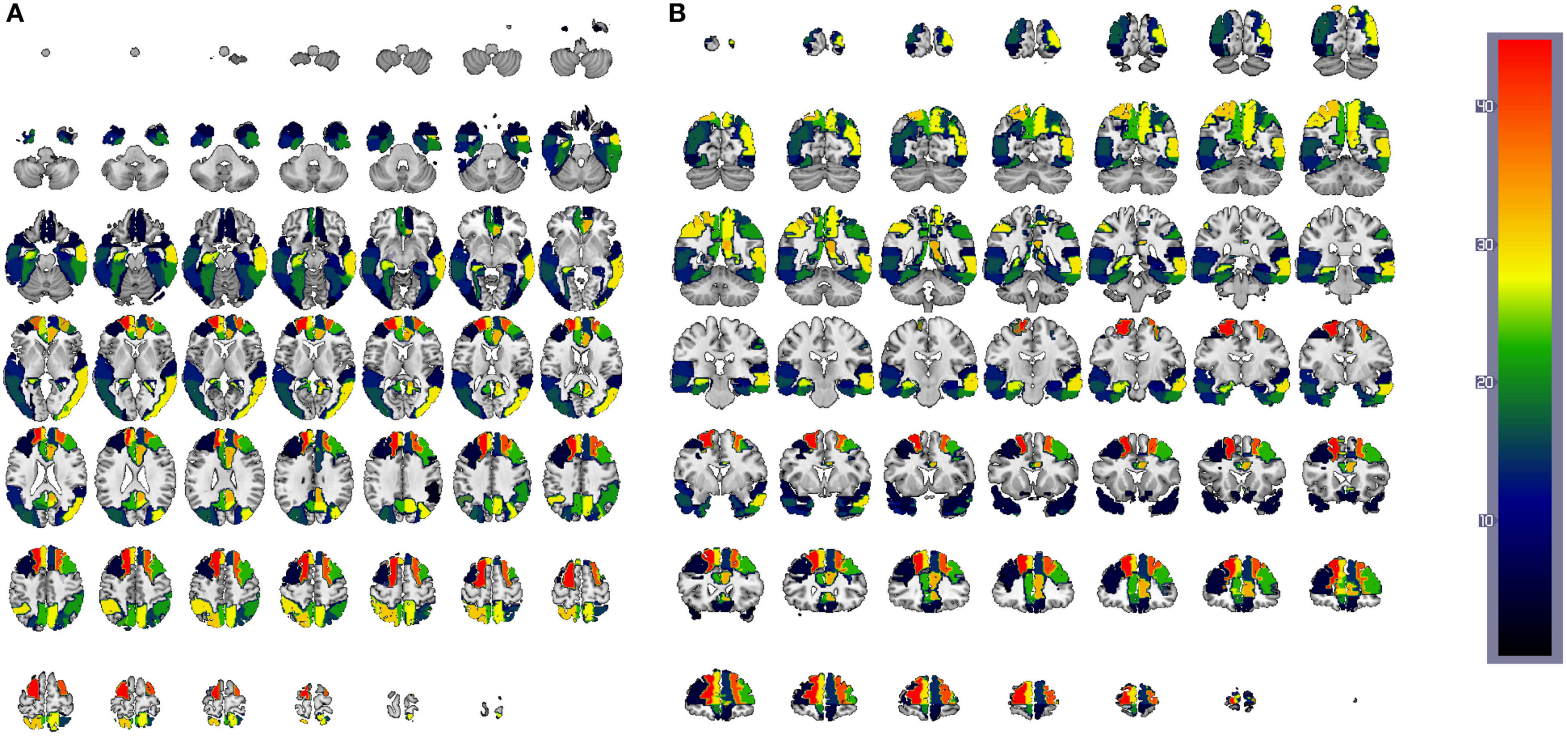
**CN/AD ROIs computed using the reconstruction error weighting for PET data**. Colors indicate the discriminative power of each ROI, which has been rescaled to the [1, 42] range. Axial **(A)** and coronal **(B)** planes are shown.

**Figure 13:**
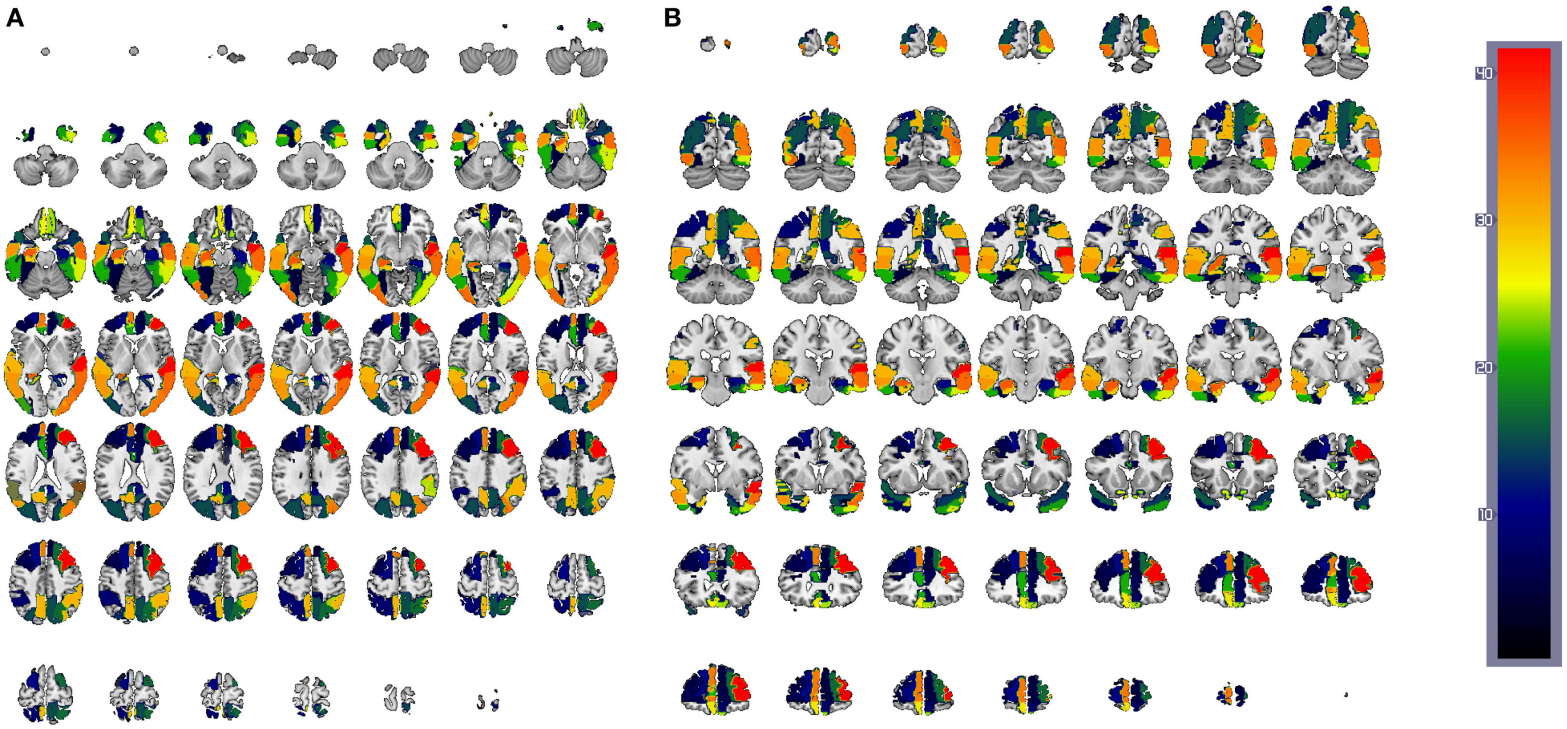
**CN/AD ROIs computed using the reconstruction error weighting for GM data**. Colors indicate the discriminative power of each ROI, which has been rescaled to the [1, 42] range. Axial **(A)** and coronal **(B)** planes are shown.

Since SVMs are trained with the residuals computed for both CN and AD models (or MCI when applied), the feature space is composed of 42 × 2 = 84 features in such a way that feature 1 and 43 correspond to the residuals obtained when regressing the mean activation level of region 1 using CN and AD models, respectively. Hence, the weight corresponding to the region *i* is computed by summing up the SVM weights for region 1 obtained with CN and AD models: $W_{1}=W_{1}^{CN}+W_{1}^{AD}$. Finally, the weight *W* is rescaled to the range [1, 42] to rank the regions. The results are shown, using a colorbar, in Figures 12, 13 for PET and MRI-GM structural SICEs, respectively. In particular, Figure 12 shows discriminative regions in the frontal-temporal lobe for PET data, indicating differences in the connectivity of these regions between CN and AD subjects, matching with ROIs found in the medical literature (Minoshima et al., 1997; Ng et al., 2004) and also with regions found by connectivity analysis (Huang et al., 2010). On the other hand, ROIs computed from GM structural data, Figure 13, show the most discriminative regions in the temporal lobe, specially in the entorhinal area (Brodmann area 28) affecting the hippocampus. These regions appear to be linked to AD in the medical literature (Nestor et al., 2004) when brain structures are analyzed using MRI.

## 4. Discussion

In this work, Gaussian graphical models have been used to work out an exploratory view of the brain network using the Sparse Inverse Covariance Matrix (computed with SICE). This allows us to build a graphical view of the brain connectivity, by modeling the inter-regional brain network.

The method presented here provides two complementary views of the brain network by means of Gaussian graphical models. The first uses the SICE method to build a functional connectivity model from FDG-PET data, which aims to reveal connectivity patters associated to Controls, MCI, and AD subjects. The second uses the SICE method on GM density data to discover inter-regional covariation of GM density. These two perspectives allow to find patterns in brain functionality and structure of CN, MCI, and AD subjects. To gain insight into these patterns, the strength of their connections is also analyzed. To reinforce the confidence in these strength data, direct values of the estimated inverse covariance matrix are compared with those provided by a devised method based on the weighted sum of the binarized inverse covariance matrices. This approach also revealed additional patterns in both, functional and structural networks. In fact, one of the main findings of this work is the variation of the connection strengths between CN, MCI, and AD subjects.

On the other hand, partial correlation coefficients obtained from the Sparse Inverse Covariance Matrix for CN and AD (or MCI) are used to build two multiple linear regression models, and subsequently, to compute the reconstruction error associated to each brain region. These reconstruction errors are used to train a SVM. Thus, applying the multiple linear regression models to new images and using the computed residuals as inputs for the trained SVM, new subjects can be classified. Indeed, the classification method proposed in this work provides classification accuracies of 92, 84, and 86%, along with AUCs of of 0.98, 0.88, and 0.91 for CN/AD, MCI/AD, and CN/MCI, respectively. The classification method also makes possible to compute regions of interest associated to AD by means of the SVM residuals, figuring out brain regions that match with regions that appear in the medical literature obtained from the analysis of FDG-PET and structural MRI image analysis.

The main findings of the paper are discussed next. We pay special attention to MCI since, while abundant literature exists in studying the differences between AD and CN, studies on MCI are limited.

- Different patterns for CN, MCI, and AD are statistically confirmed from inverse covariance matrices computed using the SICE method and functional data. When the number of arcs increases the comparisons become less relevant because weaker connections are included. Having this into account, if inter-lobe and intra-lobe connections are analyzed, we find that intra-lobe connections of the temporal lobe could be an indicator of the neurodegenerative process in MCI patients. While others, although relevant for AD, such as intra-lobe connections in the frontal lobe, do not seem so affected yet in MCI patients. When represented as graphs, CN connectivity network exhibits a good balance between integration and segregation, while MCI and AD networks are less segregated, confirming their drift away from the ideal small-worldness. The results obtained regarding to the relationship between AD-related regions match with previous results obtained with both SPECT and PET data Chaves et al. (2011, 2012).
- Although information obtained from structural data is different from that obtained for functional data, covariance inverse matrices from structural data also reveal a stronger intra-lobe covariation of GM density than inter-lobe covariation.
- Absolute values computed from SICE can be directly used as a measure of the strength of the connections. Arguing that SICE is better at detecting zero entries than giving exact values, previous works (Huang et al., 2009, 2010) only used binarized matrices. However, we prove here that computed absolute values lead to consistent results to those obtained when binarized matrices and the monotone property are combined.
- The strength of the connections reveals important information that cannot be disregarded. The importance of taking into account the strength of the connections becomes particularly clear when the graphs from structural data are analyzed. Graph metrics (clustering coefficient and average number of connections) do not show significant differences between the three groups. This is also checked from the visual inspection of the graph for 25 arcs: nodes are edges are basically the same for the three groups and the strength of the connections represents the main difference between the groups. These differences in the connection strengths are relevant when used for classification. SICE regression from structural data provides good results for the early diagnosis (CN/MCI classification).
- Classification experiments prove the discriminative capabilities of the features extracted from the inverse covariance.
- The results show covariation patterns in AD-related regions. Furthermore, high sensitivity values achieved in the classification task figure out that CN condition is easier to characterize than AD due to possible variability present in fewer atypical AD subjects. This variability is more noticeable in the MCI group. Moreover, clinical labels assigned to each patient (i.e., patients are labeled in the ADNI database using the MMSE score) have low specificity, which means low capacity to differentiate AD from other dementias such as Lewy dementia or fronto-temporal dementia.

The tool presented in this research could also have future clinical relevance to study other brain alterations and provides reliable results with small sample sizes, which is usual in clinical trials. That is, the proposed modeling and analysis procedure on AD, MCI, and CN groups can be applied to groups given a certain drug to infer differences in the connectivity models. These can also be used for longitudinal analysis, where connectivity models can be extracted for the same subject at different times. This would allow to check the evolution of the connectivity networks. It is important to note that biomarker based studies focus on individual brain regions, and therefore they can be complemented by analysis that characterize the interactions between brain regions. Finally, the classification method could also be used for the early diagnosis of AD since it has proved good performances in the first experiments with CN and MCI subjects.

### Conflict of interest statement

The authors declare that the research was conducted in the absence of any commercial or financial relationships that could be construed as a potential conflict of interest.
